# Impact of admission and early persistent stress hyperglycaemia on clinical outcomes in acute pancreatitis

**DOI:** 10.3389/fendo.2022.998499

**Published:** 2022-10-07

**Authors:** Xinmin Yang, Na Shi, Linbo Yao, Wenhua He, Ping Zhu, Sheyu Li, Lan Li, Yuying Li, Shiyu Liu, Lihui Deng, Tao Jin, Tingting Liu, Nonghua Lu, John A. Windsor, Robert Sutton, Yin Zhu, Qing Xia, Wei Huang

**Affiliations:** ^1^ West China Centre of Excellence for Pancreatitis, Institute of Integrated Traditional Chinese and Western Medicine, West China-Liverpool Biomedical Research Centre, West China Hospital, Sichuan University, Chengdu, China; ^2^ Department of Gastroenterology, First Affiliated Hospital of Nanchang University, Nanchang, China; ^3^ Department of Endocrinology and Metabolism, West China Hospital, Sichuan University, Chengdu, China; ^4^ Department of Guideline and Rapid Recommendation, Cochrane China Center, MAGIC China Centre, Chinese Evidence-Based Medicine Center, West China Hospital, Sichuan University, Chengdu, China; ^5^ Applied Surgery and Metabolism Laboratory, School of Biological Sciences, University of Auckland, Auckland, New Zealand; ^6^ Liverpool Pancreatitis Research Group, Liverpool University Hospitals National Health Service (NHS) Foundation Trust and Institute of Translational Medicine, University of Liverpool, Liverpool, United Kingdom

**Keywords:** acute pancreatitis, blood glucose, stress hyperglycaemia, hypertriglyceridaemia, clinical outcomes

## Abstract

**Background:**

To determine the impact of glucose levels at admission and during first week (early phase) on clinical outcomes in patients with acute pancreatitis (AP) and to investigate the relationship between stress hyperglycaemia (SHG) and hypertriglyceridaemia (HTG).

**Methods:**

Two independent and prospective databases were retrospectively analysed (n = 1792). Patients admitted with pain of less than 48 hours and confirmed AP were included. SHG was defined as admission blood glucose ≥ 10.00 mmol/L (non-diabetic) or ≥ 16.67 mmol/L (diabetic). Blood glucose records for the first week were inspected to determine whether SHG lasted ≥ 48 hours (persistent) or < 48 hours (transient). Clinical outcomes were compared between designated patient groups using multivariate and trend analyses. The correlation between SHG and HTG (serum triglyceride ≥ 5.65 mmol/L) was also analysed.

**Results:**

On admission, SHG was present in 27.8% (499/1792) patients; during the first 48 hours of admission, transient and persistent SHG was found in 31% (556/1792) and 8.0% (144/1792) patients, respectively. Admission SHG was associated with higher incidence of persistent organ failure, acute necrotic collection, major infection, and mortality as well as prolonged length of hospital stay (all *P* < 0.05). Duration of SHG was also associated with worsened clinical outcomes (all *P* < 0.05). In HTG-AP patients, more severe clinical outcomes were observed in those who concomitantly had SHG (*P* < 0.05).

**Conclusions:**

Admission and persistent SHG during the first week of admission worsens clinical outcomes of AP patients. These effects are more pronounced when admission HTG co-existed.

## Introduction

Acute pancreatitis (AP) is one of the leading acute gastrointestinal diseases which has no effective and targeted drug treatment (1) and causes a significant social-economic burden (2). The global incidence of AP is increasing (3) with gallstones and alcohol excess being the most common aetiologies (4). Hypertriglyceridaemia (HTG) has become more common worldwide (5) and has become one of the leading causes in China (6–8). The sequelae of AP, including diabetes mellitus (DM) has a serious impact on quality of life (9, 10). About 20% of patients with AP will develop DM within 3 years of discharge from hospital and the risk increases over time (11, 12). Early diagnosis of hyperglycaemia and optimisation of in-hospital management may help prevent AP-related DM inferred from strong evidence of critically illness (13).

It is now well known that acute illness or injury can result in hyperglycaemia, insulin resistance and glucose intolerance, collectively termed stress hyperglycaemia (SHG) (14). SHG is a key risk factor for incident DM in survivors of critical illness (13). It is plausible, however, that critical illness uncovers latent insulin resistance and/or impaired pancreatic β-cell function, such that SHG identifies patients at increased risk of subsequently developing DM (15). Evidence to date also demonstrates that prolonged severe SHG is associated with a significantly elevated risk of mortality in patients in intensive care unit (ICU) (16). While a pilot study from New Zealand suggested elevated admission fasting blood glucose (BG) might be associated with worse clinical outcomes in AP (17), the relationship between SHG and AP warrants further study. Furthermore, the relationship between SHG and HTG is uncertain. DM, dyslipidaemia and their treatment are highly linked (18, 19). Insulin treatment has been frequently used in the management of HTG-associated AP (HTG-AP), in patients both with and without diabetes (20). And while the overall clinical outcomes are worse with HTG-AP than other aetiologies (6), it is not known whether this is also attributed by SHG.

We have recently investigated the specific BG levels that define SHG in AP patients with or without pre-existing DM (21). It was found that BG ≥ 10.00 mmol/L (180 mg/dL; in non-diabetic patients) or ≥ 16.67 (300 mg/dL; in diabetic patients) were independently associated with persistent organ failure. These findings have not been validated and SHG was only investigated at the time admission without knowing the impact of SHG duration during the early phase of AP on the clinical outcomes. The aims of this study were to (1) validate and explore the impact of admission SHG and persistent SHG during first week, respectively; and (2) investigate the relationship between SHG and HTG and impact on clinical outcomes in AP patients.

## Methods

### Study design and patient population

The present study was based on the retrospective analysis using the STROBE guidelines (22) of two large Chinese AP prospective databases of consecutively enrolled AP patients. The two cohorts included patients admitted to the West China Hospital of Sichuan University (Chengdu) from January 2016 to August 2017 and First Affiliated Hospital of Nanchang University (Nanchang) from January 2011 to December 2018 (23, 24), respectively. The institutional review boards at both centres (database approval number: Chengdu, No. 2015[247]; Nanchang, No. 2011[001]) approved the study. Data of 1792 patients with AP were used in the study, 688 patients were contributed by Chengdu, and 1104 by Nanchang, respectively (**Supplementary Figures 1A, B** **)**.

### Data collection

All patients followed uniform diagnostic criteria for AP according to Revised Atlanta Criteria (RAC) (25). Comprehensive clinical data were prospectively recorded on admission, within 24 hours, 24-48 hours, day 3 and if still being hospitalised, then on days 5, 7 and once a week then as previously reported (6, 8, 21, 23, 24). The data collected included demographics of age, sex, body mass index (BMI), date of admission, time from abdominal pain onset to hospital admission, referral status, and co-morbidities. Clinical data included vital signs, haematology, biochemistry, blood gas analysis, clinical severity scores, CT pancreatic imaging. Treatment data included drugs, drainage, debridement, organ failure support. Outcome data included clinical outcomes (below), date of discharge or death. Patients were managed according to the International Association of Pancreatology/American Pancreatic Association (IAP/APA) (26).

### Inclusion and exclusion criteria

The inclusion criteria were adult patients (18-80 years) who had pain for 48 hours or less prior to admission, including those referred from other hospitals. The BG levels were determined at the time of admission and during the first week of admission at a frequency of at least every 48 hours.

The exclusion criteria were admission hypoglycaemia (BG level < 3.9 mmol/L) (27); use of glucocorticoids before admission; pregnancy or lactation; AP aetiologies of trauma, chronic pancreatitis or neoplasia; advanced comorbidities (congestive heart failure 3-4 or unstable coronary heart disease, end stage lung diseases, chronic kidney disease stage 4-5, liver cirrhosis with modified Child-Pugh grade 2-3, malignancy or immune deficiency); and incomplete data.

### Definitions

Blood glucose: admission BG data used in this study were derived from the first blood biochemistry analysis of patients who presented at emergence department and very few were obtained at general ward. Subsequent daily BG levels during the first week were from the biochemistry analysis of blood drawn prior to any potential food intake in the morning (fasted at least for 8 hours overnight).

Stress hyperglycaemia: defined as BG ≥ 10.00 mmol/L or ≥ 16.67 mmol/L for non-diabetic and diabetic patients, respectively (21), regardless of insulin treatment status.

Persistent SHG: defined as SHG not resolved after 48 hours of treatment. Patients with early fulminant pancreatitis who died within 48 hours after admission and hence were not able to develop SHG lasting > 48 hours, were included in the group of patients with persistent SHG (28, 29).

Transient SHG: defined as SHG of less than 48 hours duration regardless of insulin treatment.

Pre-existing DM: diagnosed based on disease and medicine history, or serum glycated haemoglobin (HbA1c; ≥ 6.5% or 48 mmol/mol) as per American Diabetes Association (ADA) criteria (27).

HTG-AP: defined as AP with serum triglyceride (TG) levels ≥ 5.65 mmol/L on admission after ruling out common aetiologies (6, 21). Definitions for other aetiologies were as previously described (6, 8, 21, 23, 24).

### Outcomes

The primary clinical outcome was persistent organ failure, defined as at least one of the systems (respiratory, circulatory, or renal) having modified Marshall organ failure score ≥ 2 and lasting ≥ 48 hours (25). Secondary outcome measures included multiple organ dysfunction syndrome (MODS) (2 or more systems), acute necrotic collection (25), major infection (presence of infected pancreatic necrosis, sepsis and/or pneumonia) with microbiological and/or imaging evidences (30), mortality followed up for 3 months, and length of hospital stay (LOHS).

### Statistical analysis

Continuous data are displayed as medians with 25^th^-75^th^ percentile and compared using Mann–Whitney *U* test, Kruskal-Wallis *H* test, or Cuzick’s trend test analyses. Categorical data are expressed as number with percentages and compared using Chi-square test (or Fisher’s test), linear trend test or proportional trend test analyses.

Multivariate logistic regression analysis was used to report categorical outcome measures and expressed as odds ratios (ORs) with 95% confidence intervals (CIs). Cox proportional hazards analysis was used to report LOHS and expressed as hazard ratios (HRs) with 95% CI (days for deceased patients were considered as truncated data). In both multivariate logistic regression and Cox proportional hazards analyses, baseline factors including age, or those of important clinical significance were adjusted. To quantify the effect of unmeasured potential confounding factors, we report the E-value, which represents the minimum strength of association on the risk ratio scale that an unmeasured confounder would need to have with both the exposure (with SHG) and the outcomes to fully explain away an association between the two (31). Survival differences between the duration of SHG were performed using the log rank test and plotted on Kaplan–Meier curve and adjusted using the methods of marginal balancing in groups. A two-sided *P* < 0.05 was considered statistically significant. Statistical analyses were performed using SPSS^®^ 26.0 (IBM, Armonk, New York, USA). The E-value and 95% CI were calculated using an online calculator: https://www.evalue-calculator.com/evalue/ (31, 32).

## Results

The demographics and clinical outcomes are shown in **Supplementary Table 1.** Of the included 1792 patients, the median age was 47 years (38–57), with 1135 (63.3%) were male and 245 (13.7%) had pre-existing DM. Persistent organ failure developed in 373 (20.8%) and 81 (4.5%) were MODS. Of 1510 patients had CT scan, 419 (27.7%) had acute necrotic collection. Major infection was diagnosed in 141 (7.9%) of all patients and 43 died (2.4%), all occurred in those who had persistent organ failure/MODS. The overall median LOHS was 9 days (6–14). We found these two cohorts had the same distribution of the primary outcome persistent organ failure and other important clinical outcomes such as MODS, acute necrotic collection, major infection and mortality (all *P* > 0.05) of the present study design, albeit with the demographic, aetiologies, and admission clinical severity scoring systems varied.

### SHG on admission and impact on clinical outcomes

SHG on admission was present in 499 (27.8%) patients (**Table 1**). There were no statistical differences in age, gender, pre-existing DM and time to admission between those with and without SHG. There were significant differences in BMI, Charlson comorbidity index, tertiary cases, admission TG, HTG-associated aetiology (37.3% vs 23.2%), and clinical severity scores (all *P* < 0.05) between patients with and without SHG.

**Table 1:** Baseline characteristics of patients with or without stress hyperglycaemia on admission.

	Total (n = 1792)	No stress hyperglycaemia (n = 1293)	Stress hyperglycaemia (n = 499)	*P*
Age, years, median (25^th^-75^th^ percentile)	47 (38-57)	47 (39-58)	46 (38-54)	0.094
Sex, male, n (%)	1135 (63.3)	807 (62.4)	328 (65.7)	0.191
BMI, median (25^th^-75^th^ percentile)	24.24 (22.03-26.86)	24.03 (21.8-26.37)	25.16 (22.77-27.68)	**<0.001**
Charlson comorbidity index, median (25^th^-75^th^ percentile)	0 (0-1)	0 (0-1)	0 (0-2)	**0.003**
Pre-existing DM, n (%)	245 (13.7)	176 (13.6)	69 (13.8)	0.905
Referral, n (%)	907 (50.6)	627 (48.5)	280 (56.1)	**0.004**
Time to admission, days, median (25^th^-75^th^ percentile)	1 (1-2)	1 (1-2)	1 (1-2)	0.17
Aetiology, n (%)				
Biliary	696 (38.8)	543 (42.0)	153 (30.7)	**<0.001**
HTG-associated	486 (27.1)	300 (23.2)	186 (37.3)	**<0.001**
Alcohol excess	161 (9.0)	118 (9.1)	43 (8.6)	0.736
Others or unknown	449 (25.1)	332 (25.7)	117 (23.4)	0.329
Admission glucose and lipid levels, median (25^th^-75^th^ percentile)				
Blood glucose, mmol/L	8.06 (6.38-11.61)	7.08 (5.9-8.5)	12.99 (11.24-17.25)	**<0.001**
Triglycerides, mmol/L	2.65 (1.04-10.84)	2.77 (0.95-8.24)	6.05 (1.52-14.82)	**<0.001**
Admission clinical severity scores, median (25^th^-75^th^ percentile)				
SIRS	2 (1-2)	1 (1-2)	2 (1-3)	**<0.001**
APACHE II	6 (3-8)	5 (3-8)	7 (4-10)	**<0.001**

P for Mann–Whitney U test or Chi-square test of comparison between no stress hyperglycaemia and stress hyperglycaemia groups.

BMI, body mass index; DM, diabetes mellitus; HTG, hypertriglyceridaemia; SIRS, Systemic Inflammatory Response Syndrome; APACHE II, Acute Physiology and Chronic Health Evaluation II. Bold values indicates that the P value is statistically significant.

Results for comparing clinical outcomes between patients with and without admission SHG are shown in **Table 2**. These were adjusted for baseline parameters including age, gender, BMI, Charlson comorbidity index, time to admission, referral status, biliary aetiology, admission TG levels and APACHE II score. Patients with admission SHG had significantly worse clinical outcomes: persistent organ failure (OR 2.00, 95% CI 1.51-2.65), acute necrotic collection (OR 1.78, 95%CI 1.39-2.29), major infection (OR 2.22, 95%CI 1.52-3.24), and mortality (OR 2.11, 95%CI 1.04-4.29) (all adjusted *P* < 0.05), corresponded to E-values of 2.18, 2.00, 3.87 and 3.64, respectively; the respective E-values for the low 95% CIs were 1.76, 1.64, 2.41 and 1.24. Admission SHG was also significant associated with increased LOHS (HR 0.77, 95%CI 0.68-0.85) when compared with those without SHG, E-value with the high 95% CI were 1.92 and 1.63.

**Table 2:** Comparison of clinical outcomes with or without stress hyperglycaemia on admission.

Parameters	No stress hyperglycaemia(n=1293)	Stress hyperglycaemia(n=499)	*P*	Estimate, OR or HR (95% CI)	Adjusted *P*
Persistent organ failure, n (%)	203 (15.7)	170 (34.1)	**<0.001**	2.00 (1.51-2.65)^a^	**<0.001**
MODS, n (%)	40 (3.1)	41 (8.2)	**<0.001**	1.18 (0.68-2.06)^a^	0.558
Acute necrotic collection, n (%)	245 (18.9)	174 (34.9)	**<0.001**	1.78 (1.39-2.29)^a^	**<0.001**
Major infection, n (%)	69 (5.3)	72 (14.4)	**<0.001**	2.22 (1.52-3.24)^a^	**<0.001**
Mortality, n (%)	17 (1.3)	26 (5.2)	**<0.001**	2.11 (1.04-4.29)^a^	**0.039**
LOHS, days, median (25^th^-75^th^ percentile)	8 (6-13)	11 (7-17)	**<0.001**	0.77 (0.68-0.85)^b^	**<0.001**

a Logistic regression with OR after adjusting baseline variates.

b Cox proportional hazards model with HR (deceased patients removed) after adjusting baseline variates. These variates included age, gender, body mass index, Charlson comorbidity index, time to admission, referral status, biliary aetiology, admission triglyceride levels and APACHE II that each with considerable clinical importance.

OR, odds ratio; HR, hazard ratio; CI, confidence interval; MODS, Multiple Organ Dysfunction Syndrome; LOHS, length of hospital stays. Bold values indicates that the P value is statistically significant.

### Duration of SHG and impact on clinical outcomes

Transient SHG was found in 556 (31%) and persistent SHG in 144 (8.0%) patients (139 were non-diabetic) and comparison between these two groups is demonstrated in **Supplementary Table 2**. There were no significant differences between these groups for age, gender, and alcohol aetiology (all *P* > 0.05). In patients with increased duration of SHG there was a significant increase in BMI, HTG aetiology, admission BG and TG levels, and clinical severity scores (all *P* < 0.001).

**Figure 1:**
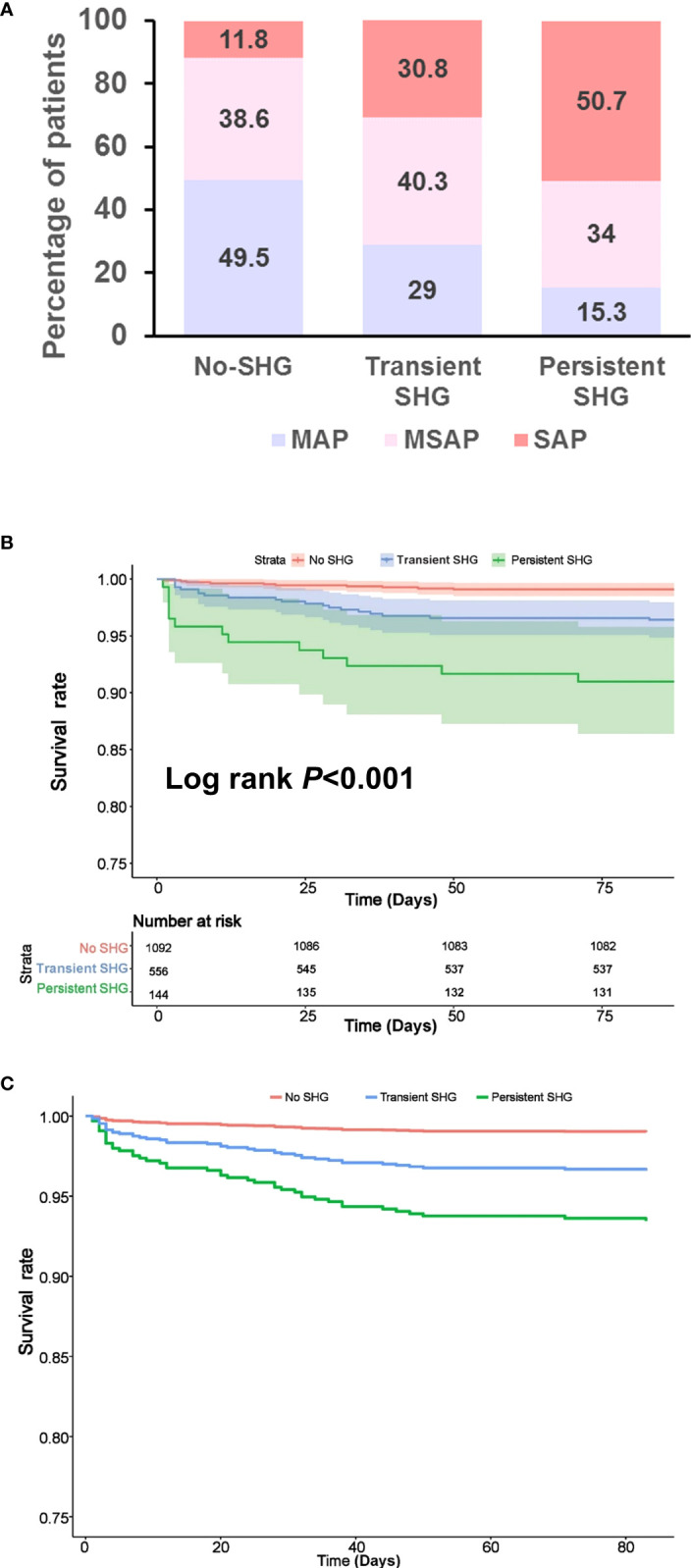
Features of patients stratified by duration of stress hyperglycaemia during the first week of admission. **(A)** Severity classification. **(B)** Kaplan–Meier survival curve. **(C)** Survival curve after adjusting for age, gender, BMI, Charlson comorbidity index, time to admission, referral status, biliary aetiology and admission TG levels. SHG, stress hyperglycaemia; MAP, mild acute pancreatitis; MSAP, moderately severe acute pancreatitis; SAP, severe acute pancreatitis.

Persistent SHG was associated with more severe AP compared with transient SHG or no SHG groups (both *P* < 0.001). There were no significant differences between transient SHG and no SHG groups (**Figure 1A**). There were 10 (0.9%), 20 (3.6%) and 13 (9.0%) deaths in no, transient and persistent SHG patients, respectively, followed a significant step-wise increase with duration of hyperglycaemia (all Log rank *P* < 0.001; **Figure 1B**), even after adjusting for baseline parameters including age, gender, BMI, Charlson comorbidity index, time to admission, referral status, biliary aetiology, and admission TG levels (all *P* < 0.001; **Figure 1C**).

**Figure 2:**
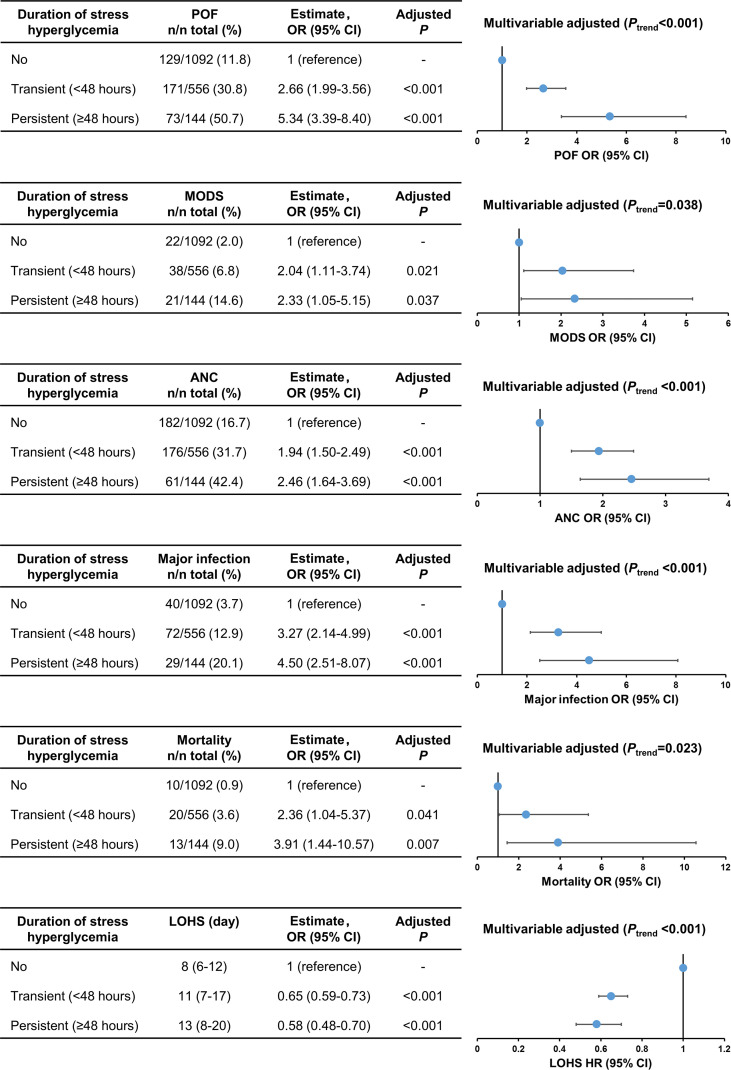
Trend analysis for clinical outcomes stratified by duration of stress hyperglycaemia. POF, persistent organ failure; MODS, Multiple Organ Dysfunction Syndrome; ANC, acute necrotic collection; LOHS, length of hospital stays.

The comparison of clinical outcomes between patients with no, transient, and persistent SHG was adjusted age, gender, BMI, Charlson comorbidity index, time to admission, referral status, biliary aetiology, admission TG levels, and APACHE II using multivariate analysis (adjusted OR or HR). Across these 3 groups there was a step-wise increase in the incidence of persistent organ failure, MODS, acute necrotic collection, major infection, and mortality with corresponding prolonged LOHS (all *P*_trend_ < 0.05; **Figure 2**). In addition, the clinical outcomes were worse in patients with persistent SHG compared with those with admission SHG (**Supplementary Figure 2**).

### Relationship between SHG and HTG and impact on clinical outcomes

There was a significant positive association between admission BG and TG levels (*r_s_* = 0.297, *P* < 0.001; **Supplementary Figure 3A**). The incidence of SHG in AP patients with HTG was significantly higher than those with non-HTG (39.2% vs 21.3%, *P* < 0.001; **Supplementary Figure 3B**).

Subgroup analyses compared the impact of admission SHG on clinical outcomes between non-HTG and HTG-associated AP patients. The clinical outcomes were worse in non-HTG-AP patients with admission SHG than those without SHG (**Table 3**, *upper panel*). The same was true for HTG-AP patients (**Table 3**, *lower panel*). These results were confirmed after adjusting for age, gender, BMI, Charlson comorbidity index, time to admission, and referral status (**Table 3**).

**Table 3:** Clinical outcomes of patients stratified by stress hyperglycaemia in non-HTG and HTG groups on admission.

Non-HTG patients (n = 1139)					
Parameters	No stress hyperglycaemia(n = 896)	Stress hyperglycaemia(n = 243)	*P*	Estimate, OR or HR (95% CI)	Adjusted *P*
Persistent organ failure, n (%)	136 (15.2)	78 (32.1)	**<0.001**	2.47 (1.76-3.47)^a^	**<0.001**
MODS, n (%)	25 (2.8)	9 (3.7)	0.458	1.18 (0.53-2.63)^a^	0.695
Acute necrotic collection, n (%)	156 (17.4)	87 (35.8)	**<0.001**	2.54 (1.84-3.51)^a^	**<0.001**
Major infection, n (%)	45 (5.0)	38 (15.6)	**<0.001**	3.28 (2.06-5.23)^a^	**<0.001**
Mortality, n (%)	10 (1.1)	11 (4.5)	**<0.001**	3.76 (1.52-9.31)^a^	**0.004**
LOHS, days, median (25^th^-75^th^ percentile)	8 (6-12)	10 (6-16)	**0.001**	0.77 (0.61-0.83)^b^	**<0.001**
**HTG-associated patients (n = 653)**					
**Parameters**	**No stress hyperglycaemia** **(n = 397)**	**Stress hyperglycaemia** **(n = 256)**	*P*	**Estimate,** **OR or HR (95% CI)**	**Adjusted *P***
Persistent organ failure, n (%)	67 (16.9)	92 (35.9)	**<0.001**	2.51 (1.71-3.68)^a^	**<0.001**
MODS, n (%)	15 (3.8)	32 (12.5)	**<0.001**	2.88 (1.48-5.60)^a^	**0.002**
Acute necrotic collection, n (%)	89 (22.4)	87 (34.0)	**0.001**	1.55 (1.07-2.22)^a^	**0.019**
Major infection, n (%)	24 (6.0)	34 (13.3)	**0.002**	2.12 (1.21-3.72)^a^	**0.009**
Mortality, n (%)	7 (1.8)	15 (5.9)	**0.005**	2.76 (1.08-7.04)^a^	**0.034**
LOHS, days, median (25^th^-75^th^ percentile)	9 (7-13)	12 (8-19)	**<0.001**	0.72 (0.61-0.85)^b^	**<0.001**

a Logistic regression with OR after adjusting baseline variates.

b Cox proportional hazards model with HR (deceased patients removed) after adjusting baseline variates. These variates included age, gender, body mass index, Charlson comorbidity index, time to admission and referral status that each with considerable clinical importance.

HTG, hypertriglyceridaemia; OR, odds ratio; HR, hazard ratio; CI, confidence interval; MODS, Multiple Organ Dysfunction Syndrome; LOHS, length of hospital stay. Bold values indicates that the P value is statistically significant.

## Discussion

In this study, we have validated the newly defined SHG in AP patients showing a step-wise relationship between increased admission glucose levels and worsened clinical outcomes. This study also demonstrates that AP patients can be classified according to glycaemic status during the disease early phase (first week) into no, transient, and persistent SHG which were associated with escalating disease severity and adverse clinical outcomes; clinical outcomes were worse in patients with persistent SHG compared with those with admission SHG. Furthermore, the association of SHG with adverse clinical outcomes remained robust in HTG-AP patients.

A large number of studies have focused on the probability of DM after pancreatitis and its possible mechanism (11, 12, 33–35). Few have studied on BG changes under stress status and its impact on the severity or outcomes of AP patients (21). The pathogenesis of SHG during critical illness is complex, including increased release of counter-regulatory hormones, altered insulin receptor signalling due to inflammation, pancreatic beta-cell inhibition and interventions such as administration of glucocorticoids or parenteral nutrition (36, 37). How glucose metabolism is affected by derangement of adrenaline, glucagon, cortisol, and insulin remains to be elusive due to lack of comparable studies, it is relatively clearer that hyperglycaemia in AP mainly due to both impairment of beta-cells resulting a decrease in insulin secretion and the production of cytokines. This causes the appearance/worsening of insulin resistance and subsequently induces hyperglycaemia, which, in turn, may further damage beta-cells and worsen insulin resistance observed in critical illness (16). In this study, we also further demonstrate that AP patients with SHG often had higher BMI and TG levels, suggesting that well accepted risk factors for DM also contribute to the development of SHG.

While the definition of SHG varied among studies (36, 38), the strength of the association between each glucose parameter and outcome emphasises the significance of this relationship. SHG is associated with a significantly elevated risk of mortality in ICU patients (16). A recently meta-analysis demonstrated that COVID-19 patients with hyperglycaemia (BG ≥ 7, 7.7, 10, or 11 mmol/L) also had a higher risk of developing severe or critical illness compared with normoglycaemia patients regardless of prior DM conditions (39). Similarly, we optimised SHG as BG ≥ 10.00 mmol/L and ≥ 16.67 mmol/L for AP patients without pre-existing diabetes and those with pre-existing diabetes, respectively, according to multivariate logistic regression and ROC curves) on admission (21). Here, we further verified these admission BG cut-off values and confirmed a step-wise relationship between increased glucose levels and worsened clinical outcomes in AP patients, consistent with our previous findings. Sensitivity analyses revealed that it would take very strong confounding to negate the associations observed in this study. These observations are the same when analysing the two composition cohorts of varied baseline characteristics and aetiologies separately (data not shown).

Persistent hyperglycaemia is a common parameter used to evaluate blood glucose fluctuations, and our finding of their association with adverse clinical outcomes in AP patients is similar to that in acute ischaemic stroke, acute myocardial infarction, intracerebral haemorrhage, and other critical illness (28, 29, 40, 41). On the other hand, the early phase (first week) in severe AP patients is often accompanied by persistent SIRS which develops to persistent organ failure/MODS, serving as the predominate cause of death (8, 42). Duration of organ failure during the first week had proved to be strongly associated with the risk of local complications and death (8, 43). Therefore, in the current study, we also studied dynamic nature of glucose changes and their association with clinical outcomes during the first week in AP patients. Persistent SHG was defined as the SHG persisted over 48 hours as other disease definitions (28, 29), and we newly defined transient SHG for those SHG presented but less than 48 hours referred to the definition time of whether the organ failure in AP persists or not (25). There was a stepwise increase of adverse clinical outcomes in patients with no, transient, and persistent SHG. And the increase was more pronounced with persistent SHG than with admission SHG. This means that once SHG occurred during the first week of admission, the risk of all adverse clinical outcomes increased, and the longer the duration of SHG, the more serious the clinical outcome would be. However, we cannot directly establish the cause-effect relationship claiming that SHG aggravated the severity of AP in the current study. Whether SHG worsens clinical outcomes of AP patients warrant further basic and clinical research.

HTG has been reported as the aetiology of AP in more than a third of cases in some large Chinese AP cohorts (6–8). This may be due to an increasing prevalence of central obesity (44) and/or metabolic syndrome in Chinese populations (45). We and others have previously shown that admission TG levels are associated with worse clinical outcomes in AP patients (6, 46), but these analyses did not account for co-existing SHG. We took a subgroup analysis for HTG-AP and found the similar adverse effect of SHG on the outcomes as for the whole cohort of AP. These findings highlight a close interaction between glucose haemostasis and lipid metabolism (47, 48). This is an important finding because it provides justification for strategies to lower both glucose and TG in the acute management of AP. A recent meta-analysis of observational studies supported the use of insulin for the early management of HTG-AP patients (49). Recently, a compelling experimental study suggests that endogenous insulin protected pancreatic acinar cells during AP by preserving glycolytic ATP supply to calcium pumps (50). Therefore, insulin can protect against AP by both acting on acinar cells and hormone-sensitive lipase (preventing release of free fatty acids from TG) and this warrants clinical trials.

Our study has several limitations. Firstly, as the nature of *post hoc* analysis, the BG data were not available for every day (data were mainly missing on day 4 and day 6), which may cause the lower proportion of transient SHG than it actually was. Secondly, only more than half of the patients had HbA1c measured on admission and thus we most likely underestimate the prevalence of pre-existing DM in out cohorts. Thirdly, we could not determine from both databases what the timing of and which treatment strategies affected the glucose levels. Therefore, we did not try to perform an analysis of glycaemic variability. Further research with individual-level data on treatment type and timing may help clarify these questions. Finally, we did not investigate the impact of admission and duration of SHG on the probability of developing post-pancreatitis DM, which will comprise a separate study.

## Conclusion

In conclusion, we demonstrate that the admission and duration of SHG had important impact on development adverse clinical outcomes in AP patients. Screening, monitoring, and targeting AP patients with high-risk of developing SHG may have beneficial clinical implication.

## Data availability statement

The original contributions presented in the study are included in the article/**Supplementary Material**. Further inquiries can be directed to the corresponding authors.

## Author contributions

XY and NS contributed equally to this study. XY, NS, WHe, LL, YL, LY, LD, TJ, NL, and YZ: acquisition of data. XY, NS, and WHu: drafting of manuscript. XY, NS, WHe, PZ, TL, LD, TJ, and WHu: analysis and interpretation of data. SheL and PZ: statistical analysis supervision. ShiL, RS, and JW: important intelligence input. RS, JW, YZ, and QX: critical revision of the manuscript. WHu, QX, and YZ: study concept and design, obtained funding, study supervision. All authors contributed to the article and approved the submitted version.

## Funding

This work was supported by National Natural Science Foundation of China (No. 81973632, No. 82270672, WHu; No. 81774120, QX; No. 81960128, YZ; No. 82100682, NS); Key Research and Development Programme of Science and Technology Department of Sichuan Province (2022YFS0406, XY; 2020YFS0235, NS); China Postdoctoral Science Foundation (2022M712285, XY; 2022T150453, XY); NIHR Senior Investigator Award (RS).

## Acknowledgments

These authors thank all the staff from the pancreas multidisciplinary teams at West China Hospital of Sichuan University and First Affiliated Hospital of Nanchang University for their continuous support.

## Conflict of interest

The authors declare that the research was conducted in the absence of any commercial or financial relationships that could be construed as a potential conflict of interest.

## Publisher’s note

All claims expressed in this article are solely those of the authors and do not necessarily represent those of their affiliated organizations, or those of the publisher, the editors and the reviewers. Any product that may be evaluated in this article, or claim that may be made by its manufacturer, is not guaranteed or endorsed by the publisher.

## Supplementary material

Supplementary Figure 1 Patient selection flow chart. **(A)** Chengdu cohort. **(B)** Nanchang cohort. AP, acute pancreatitis; CP, chronic pancreatitis.

Supplementary Figure 2 Adverse clinical outcomes in patients with admission and persistent stress hyperglycaemia. SHG, stress hyperglycaemia; POF, persistent organ failure; MODS, multiple organ dysfunction syndrome; ANC, acute necrotic collection.

Supplementary Figure 3 Glucose and lipid metabolism disorder on admission in AP patients. **(A)** Correlation between glucose and triglycerides. **(B)** Admission stress hyperglycaemia in HTG and non-HTG AP patients. TG, triglycerides; MAP, mild acute pancreatitis; MSAP, moderately severe acute pancreatitis; SAP, severe acute pancreatitis; No-SHG, no stress hyperglycaemia; SHG, stress hyperglycaemia.
