# Supplementary figures and images for: Impact of admission and early persistent stress hyperglycaemia on clinical outcomes in acute pancreatitis
Source: Front Endocrinol (Lausanne). 2022 Oct 7;13:998499. doi: 10.3389/fendo.2022.998499 (PMC9585288; doi:10.3389/fendo.2022.998499)

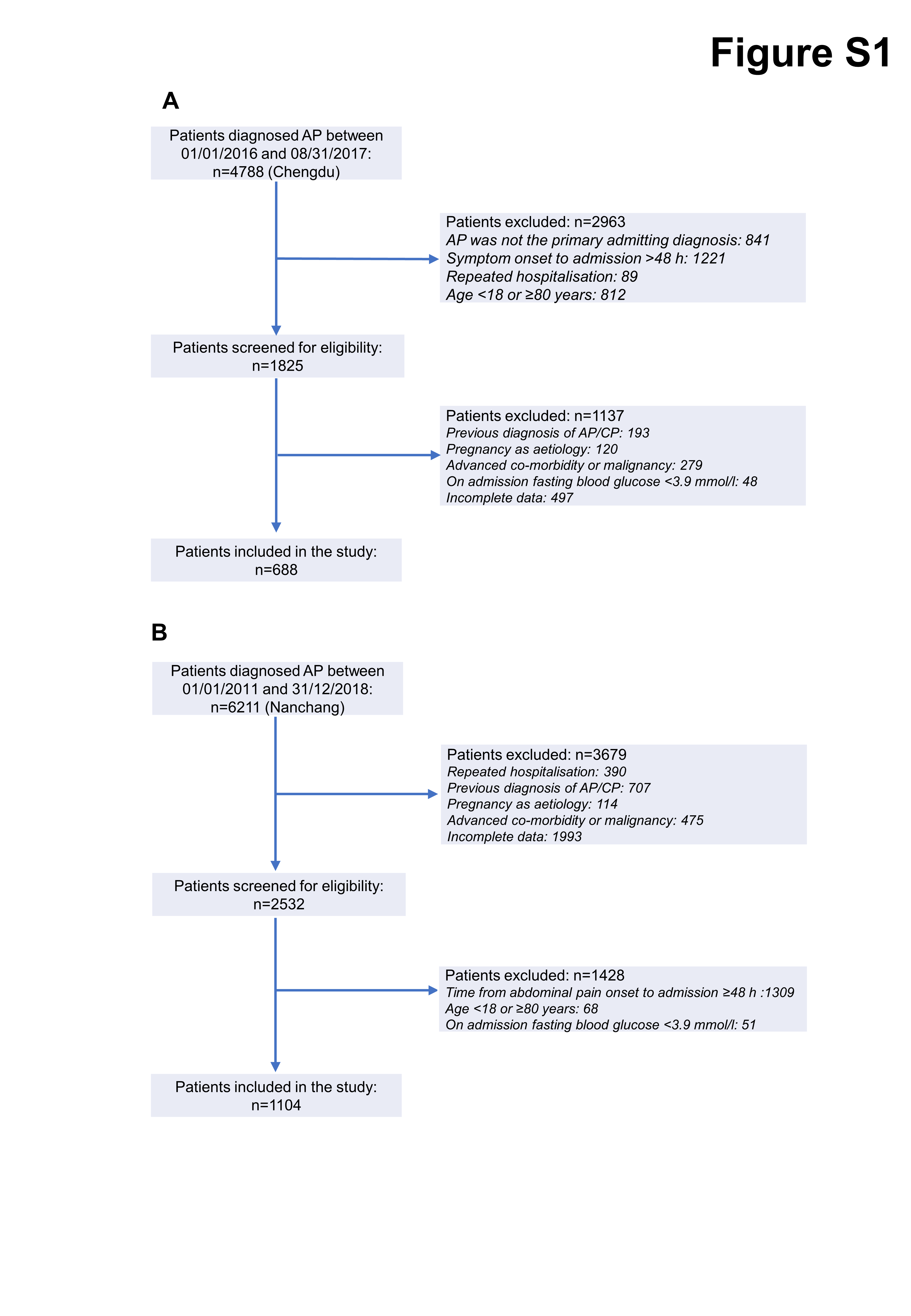

Supplement: Supplementary Figure 1 — Patient selection flow chart. (A) Chengdu cohort. (B) Nanchang cohort. AP, acute pancreatitis; CP, chronic pancreatitis. [file Image_1.tif]

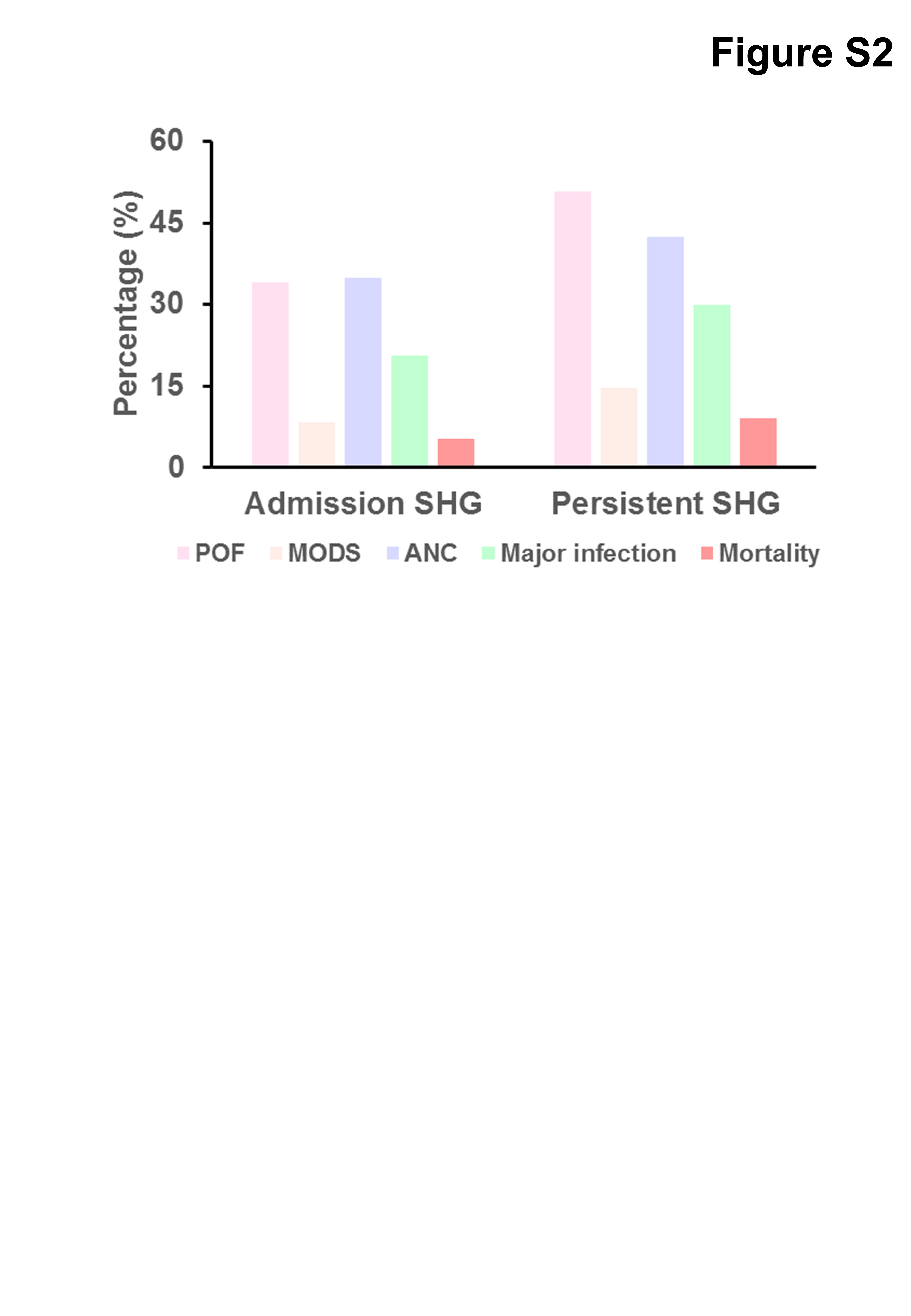

Supplement: Supplementary Figure 2 — Adverse clinical outcomes in patients with admission and persistent stress hyperglycaemia. SHG, stress hyperglycaemia; POF, persistent organ failure; MODS, multiple organ dysfunction syndrome; ANC, acute necrotic collection. [file Image_2.tif]

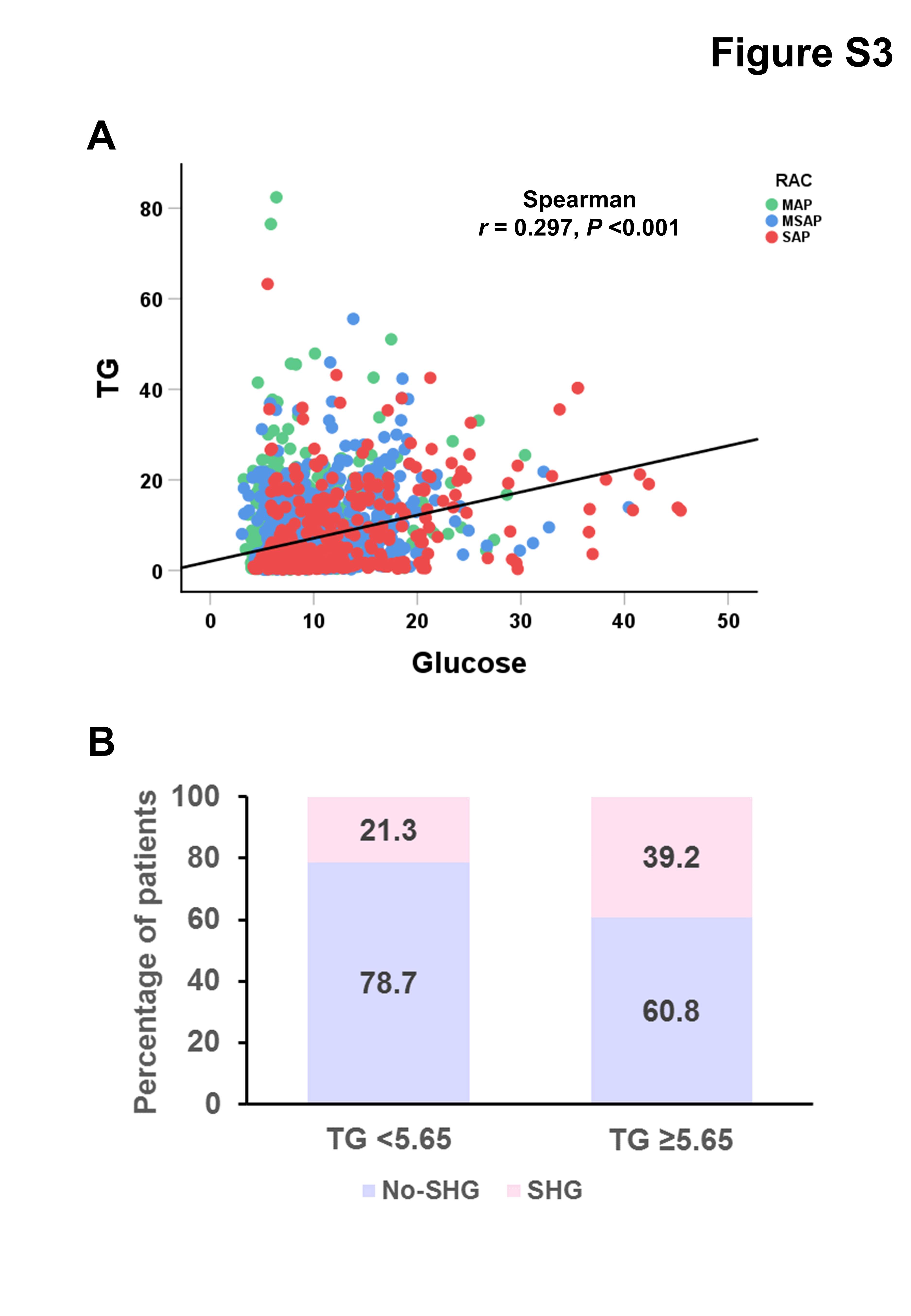

Supplement: Supplementary Figure 3 — Glucose and lipid metabolism disorder on admission in AP patients. (A) Correlation between glucose and triglycerides. (B) Admission stress hyperglycaemia in HTG and non-HTG AP patients. TG, triglycerides; MAP, mild acute pancreatitis; MSAP, moderately severe acute pancreatitis; SAP, severe acute pancreatitis; No-SHG, no stress hyperglycaemia; SHG, stress hyperglycaemia. [file Image_3.tif]
