# Supplementary material for: Impact of admission and early persistent stress hyperglycaemia on clinical outcomes in acute pancreatitis
Source: Front Endocrinol (Lausanne). 2022 Oct 7;13:998499. doi: 10.3389/fendo.2022.998499 (PMC9585288; doi:10.3389/fendo.2022.998499)
Supplement: Supplementary file 4 [file Table_1.docx]

**Table S1.** Baseline characteristics and clinical outcomes of patients from two cohorts

|  | **Total**  **(n = 1792)** | **Chengdu**  **(n = 688)** | **Nanchang**  **(n = 1104)** | ***P* value** |
| --- | --- | --- | --- | --- |
| Age, years, median (25^th^-75^th^ percentile) | 47 (38-57) | 45 (38-51) | 49 (39-62) | **<0.001** |
| Sex, male, n (%) | 1135 (63.3) | 471 (68.5) | 664 (60.1) | **<0.001** |
| BMI, median (25^th^-75^th^ percentile) | 24.24 (22.03-26.86) | 25.39 (23.09-27.70) | 23.61 (21.38-25.99) | **<0.001** |
| Charlson comorbidity index, median (25^th^-75^th^ percentile) | 0 (0-1) | 1 (0-2) | 0 (0-1) | **<0.001** |
| Pre-existing DM, n (%) | 245 (13.7) | 122 (17.7) | 123 (11.1) | **<0.001** |
| Referral, n (%) | 907 (50.6) | 383 (55.7) | 524 (47.5) | **0.001** |
| Aetiology, n (%) |  |  |  |  |
| Biliary | 696 (38.8) | 156 (22.7) | 540 (48.9) | **<0.001** |
| HTG-associated | 486 (27.1) | 276 (40.1) | 210 (19.0) | **<0.001** |
| Alcohol excess | 161 (9.0) | 50 (7.3) | 111 (10.1) | 0.045 |
| Others or unknown | 449 (25.1) | 206 (29.9) | 243 (22.0) | **<0.001** |
| Admission glucose and lipid levels, median (25^th^-75^th^ percentile) |  |  |  |  |
| Blood glucose, mmol/L | 8.06 (6.38-11.61) | 8.3 (6.45-12.39) | 7.86 (6.33-11.24) | **0.01** |
| Triglycerides, mmol/L | 2.65 (1.04-10.84) | 9.17 (1.96-17.64) | 1.64 (0.87-5.14) | **<0.001** |
| Admission clinical severity scores, median (25^th^-75^th^ percentile) |  |  |  |  |
| SIRS | 2 (1-2) | 2 (1-3) | 1 (1-2) | **<0.001** |
| APACHE II | 6 (3-8) | 4 (2-6) | 7 (4-10) | **<0.001** |
| Persistent organ failure, n (%) | 373 (20.8) | 133 (19.3) | 240 (21.7) | 0.222 |
| MODS, n (%) | 81 (4.5) | 25 (3.6) | 56 (5.1) | 0.154 |
| Acute necrotic collection, n (%) | 419 (23.4) | 144 (20.9) | 275 (24.9) | 0.053 |
| Major infection, n (%) | 141 (7.9) | 54 (7.8) | 87 (7.9) | 0.981 |
| Mortality, n (%) | 43 (2.4) | 21 (3.1) | 22 (2.0) | 0.154 |
| LOHS, median (25^th^-75^th^ percentile) | 9 (6-14) | 10 (7-15) | 8 (6-13) | **<0.001** |

*P* for Mann–Whitney *U* test or Chi-square test of comparison between Chengdu and Nanchang cohorts.

BMI, body mass index; DM, diabetes mellitus; HTG, hypertriglyceridaemia; SIRS, Systemic Inflammatory Response Syndrome; APACHE II, Acute Physiology and Chronic Health Evaluation II; MODS, Multiple Organ Dysfunction Syndrome; LOHS, length of hospital stay.
