# Supplementary material for: Impact of admission and early persistent stress hyperglycaemia on clinical outcomes in acute pancreatitis
Source: Front Endocrinol (Lausanne). 2022 Oct 7;13:998499. doi: 10.3389/fendo.2022.998499 (PMC9585288; doi:10.3389/fendo.2022.998499)
Supplement: Supplementary file 5 [file Table_2.docx]

**Table S2.** Baseline characteristics of different duration of stress hyperglycaemia groups during the first week

|  | **No** **stress hyperglycaemia**  **(n = 1092)** | **Transient hyperglycaemia**  **(n = 556)** | **Persistent hyperglycaemia**  **(n = 144)** | ***P* value** |
| --- | --- | --- | --- | --- |
| Age, years (25^th^-75^th^ percentile) | 47 (38-58) | 47 (39-55) | 45 (37-52) | 0.057 |
| Sex, male, n (%) | 681 (62.4) | 355 (63.8) | 99 (68.8) | 0.313 |
| BMI (25^th^-75^th^ percentile) | 23.94 (21.71-26.35) | 24.5 (22.33-26.89) | 26.26 (24.28-28.83) | **< 0.001** |
| Charlson comorbidity index (25^th^-75^th^ percentile) | 0 (0-1) | 1 (0-2) | 1 (0-2) | **< 0.001** |
| Pre-existing DM, n (%) | 149 (13.6) | 91 (16.4) | 5 (3.5) | **< 0.001** |
| Referral, n (%) | 511 (46.8) | 307 (55.2) | 89 (61.8) | **< 0.001** |
| Time to admission, days (25^th^-75^th^ percentile) | 1 (1-2) | 1 (1-2) | 2 (1-2) | **0.005** |
| Aetiology, n (%) |  |  |  |  |
| Biliary | 476 (43.6) | 190 (34.2) | 30 (20.8) | **< 0.001** |
| HTG-associated | 223 (20.4) | 185 (33.3) | 78 (54.2) | **< 0.001** |
| Alcohol excess | 95 (8.7) | 56 (10.1) | 10 (6.9) | 0.439 |
| Others or unknown | 298 (27.3) | 125 (22.5) | 26 (18.1) | **0.013** |
| Admission glucose and lipid levels (25^th^-75^th^ percentile) |  |  |  |  |
| Blood glucose, mmol/L | 6.99 (5.86-8.32) | 11.27 (8.97-14.02) | 15.32 (11.72-18.54) | **< 0.001** |
| Triglycerides, mmol/L | 1.95 (0.93-6.82) | 4.42 (1.3-13.51) | 11.58 (4.19-19.63) | **< 0.001** |
| Admission clinical severity scores (25^th^-75^th^ percentile) |  |  |  |  |
| SIRS | 1 (1-2) | 2 (1-3) | 2 (1.5-3) | **< 0.001** |
| APACHE II | 5 (3-8) | 6 (4-9) | 7 (4-11) | **< 0.001** |

*P* for Kruskal-Wallis *H* test and Chi-square test (or Fisher’s test) of the 3 groups.

BMI, body mass index; DM, diabetes mellitus; HTG, hypertriglyceridaemia; SIRS, Systemic Inflammatory Response Syndrome; APACHE II, Acute Physiology and Chronic Health Evaluation II.
